# Inhalation of Ethereal Vapor, for Inducing Insensibility to the Pain of Surgical Operations

**Published:** 1847-03

**Authors:** A. Hill


### ARTICLE II.

### *Inhalation of Ethereal Vapor, for the purpose of inducing Insensibility to the Pain of Surgical Operations.* By A. HILL, D. D. S.

No recent discovery seems to be exciting more interest at the present time, among professional and non-professional circles, than the discovery claimed and patented by Drs. Jackson and Morton, of Boston. The announcement of the wonderful effects produced by it, has struck the public ear with astonishment, and the public mind with delightful surprise. At the same time, it has been the signal for the most severe and unjust attacks upon its fortunate (?) discoverers. And while it has thrown a certain class into ecstasies of pleasure, from the delightful visions it has inspired in their minds, of future exemption and immunity from pain in connection with surgical operations, it seems to have had a directly opposite effect upon another class, who, so far from feeling these delightful emotions, are affected with the most violent spasms, and vent the whole force of their fury against it. Indeed, it is somewhat doubtful whether those who have taken the "*Letheon*" and those who have not, by direct application, have been most seriously affected by it. To a certain class, it is like a nauseous drug, to one who has been sickened by it, and who only needs to hear it mentioned, or that its offensive odor should strike his olfactories in the slightest manner, to excite in his stomach the most violent retchings; while another class are loud in its praises and bold in its defence. Many a noble and high minded man will, doubtless, be satisfied to condemn it *a priori*, while others, less hasty and a little less dogmatical, will insist upon a fair and impartial trial. Some, who are unworthy and unfit to apply it in any case, but who happen to be fortunate, or rather *unfortunate* enough to obtain a "license," will use it in such a manner as to bring discredit upon the whole concern. Others, again, will condemn it *in toto*, from the fact that it has been "patented," and, whatever virtues there may be in it, with them, and as far as they are concerned, will go for nothing worth, such is their abhorrence of "patent medicines," and their love of professional courtesy and conventional etiquette. Now, with all due deference to my professional brethren, and those who may differ from me upon this subject, I honestly beg leave to say that, in my opinion, there is one way, and *but* one correct way, of placing this matter in its true light and upon its proper basis, and that way is, to give the thing a fair and impartial test, note all the circumstances attending it, and let stern and rigid facts attest its truth, or demolish its claims to our consideration. That there should be strong friends and violent enemies, is to be expected from the nature of the case. And, if it accomplish one half of what at first it promised, it will nobly redeem itself from the ignominious death to which many are eager to doom it, without judge or jury, or even the "benefit of clergy."

The very announcement of such a discovery seems to take the world aback, even in this day of wonders and strange inventions. Nor is it singular; for if true, or even half true, it is stupendous, it is wonderful indeed. In such cases, when did speculation fail to run rampant? when did selfish, sordid interest fail to cry out "*humbug!*" when did blear-eyed envy fail to look askance? Certainly not since the time when the discovery of the immortal Jenner was condemned, *a priori*, and sentenced to the shades, before a trial could be had. But his discovery lived to bless the world, nevertheless, as others have done under similar circumstances. What, if its inventor should secure a patent at Washington? Is this sufficient for its utter condemnation? What if, in the hands of ignorant and unskilful men, the thing should prove a failure—nay, worse than that, an injury—must the thing be abandoned forever, and all the benefits that otherwise might accrue, be lost to our pain-stricken world? And would not such objections strike from the materia medica a very large list of our most valuable remedies? Who among us dare affirm that mercury, in one form of preparation or another, has not actually killed more than it has ever cured? and so of opium, and a thousand other things; and yet where is the man of science and skill, who wishes them all thrown overboard, at once and forever? What intelligent dental surgeon but believes that thousands of valuable teeth have been ruined, and are yearly sacrificed, to the stupid cupidity of ignorant pretenders? And yet, would he abjure the profession? No! no! Then I respectfully suggest, whether we are not at fault when we so freely, and without a patient trial, denounce the use of "Morton's Letheon," in surgical operations. With these few prefatory remarks, I proceed to detail some of my experiments with the "*Letheon.*"

Seeing the statements of such men as Drs. Warren, Hayward, Bigelow and others—men, than whom it is difficult to find higher or more reputable medical authority in this country—in reference to the effects resulting from its administration, I determined to give it a trial. Accordingly, I procured a "license" for its use, from Dr. Morton of Boston, and, with fear and trembling, commenced my experiments. The first case was that of the extraction of an inferior canine tooth, from the jaw of a gentleman about thirty years of age; the tooth was a large one, exceedingly inflamed and sore. After inhaling the vapor about one minute and a half, I proceeded to the removal of the tooth, in presence of several gentlemen, two of which were members of the dental profession. The tooth was a very large one, and came very hard, yet the patient evinced not the slightest sense of pain, although he previously flinched a good deal, as I laid my fingers on his lips to open his mouth. He continued in a sitting posture for some six or eight seconds afterwards, with his head back, and a pleasant smile upon his face, when he raised his head, spit out the blood, just as in ordinary cases, and said, "Doctor, that is first rate," and left the room delighted with the operation. I have seen him many times since, and he fails not to recommend the Letheon to his friends. This case gave me encouragement, and I carefully proceeded. Case after case occurred, with the same results; which cases I have carefully noted, and can now say, that I have more confidence in its virtues than at the first, and less apprehension of any evil effects resulting from its administration. I omitted to say, in reference to the first case mentioned, that the patient declared, again and again, *he felt no pain* during the extraction of his tooth. I have since administered the vapor to some twenty or thirty persons, of different ages, sexes and conditions, and extracted between forty and fifty teeth, while the patients were under its influence and with the following results. In nearly all cases, the patients testified they felt no pain during the operation; some four or five felt a slight pain, but nothing to be compared with the ordinary amount of suffering in such cases. About half of them were conscious of all my movements, knew what I said to them, but felt no pain. Few of them inhaled longer than two minutes—more of them from one to one and a half—several less than one minute. In *every* case where the operation was a partial failure, there was an imperfect inhalation, the patient refusing to take it according to my directions. In one case, that of a gentleman, there was considerable nausea after the inhalation. Several circumstances contributed to induce it. 1st. He came with several others, in a perfect glee. 2d. He took a large quantity of the vapor before it could be made available for the removal of his tooth, in consequenc of removing the tube from his mouth, stopping to laugh, &c. &c., and necessarily swallowed much of the gas with his saliva. After continuing in this way for some time, I remonstrated with him, when he tried again, and I removed a large inferior molar. He remarked, upon the first inhalation, that it went against his stomach, and that he disliked the smell of it; these circumstances, beyond question, were the cause of the nausea. This gradually passed off, with no other evil effects. The next morning his wife came and took the Letheon, and had two teeth removed, without the consciousness of any pain, and was perfectly delighted with the operation, and said, "she loved the gas." Several patients, after their teeth were removed, have stoutly and persistingly denied that they were out, and could only be convinced by looking at the tooth, and putting their fingers in the bleeding socket. Several patients dreamed while under its influence, and related their dreams afterwards. Some expressed themselves as being in a state of ecstatic enjoyment, while others were confused, and could not tell their feelings. In every instance, except the one above mentioned, that was nauseated, not the slightest evil effects have come to my knowledge, although I have taken much pains to inquire after their health and feelings.

A few days since, I was sent for to administer the gas in an adjoining town, to a young gentleman, about seventeen years of age, for the amputation of his leg. I found the young man very weak and much emaciated, in consequence of a large scrofulous swelling, just below his right knee. His tongue was much coated, and his pulse ranging from one hundred to one hundred and twenty, and had been for several days. The attending physicians had concluded to amputate his limb, as the only means of saving his life, and his friends, anxious to relieve his sufferings, sent for me to administer the Letheon. I explained to the lad and his friends the probable effects of the Letheon, and that its success or failure all depended upon the manner in which it was inhaled; (for I had previously found that where there was a partial failure, in *every* case, it was the result of imperfect inhalation.) Every thing being in readiness, the boy commenced the inhalation, and continued to breathe the Letheon for one minute and a half, when I informed the Dr. he could commence the amputation, and immediately removed the tube from his mouth. The first remark the lad made was, "I thought you were deceiving me, but I find I have been dreaming." This occurred just as the limb was removed. But consciousness was not fully restored until nearly at the close of the operation. After the stump was dressed, and the boy removed to his bed, I requested him, in presence of several persons, among whom were two or three physicians, to tell me what he remembered and what he felt of the operation. Said he, "I did not feel anything until the bone was off; I was dreaming." Said I, "What did you dream?" Said he, "I dreamed that you was giving me that Letheon, and would not know when to stop, and that I should choke to death."

The operation was performed by Dr. Lacy, assisted by Drs. Bennett and Booth; all of whom expressed themselves satisfied and pleased with the effects of the vapor. And I am now satisfied that the lad could have inhaled a much longer time, without the slighest injury, and probably with better success. The limb was taken off above the knee, about midway of the femoral bone. From all these circumstances, and others which have come to my knowledge, I have arrived at the following conclusions:

1st. That, in the hands of *careful*, *skilful* and *judicious* men, the Letheon is perfectly safe.

2d. That where there is a failure, it is the result of an imperfect preparation of the vapor, or an imperfect inhalation, modified, perhaps, in some cases, by peculiar temperament.

3d. That it is not only safe, but valuable beyond conception, in a multitude of cases.

That there may be some cases, where temporary injury may result, or even permanent injury, I cannot deny; yet I am by no means sure, provided the requisite care is taken.

Whether it is safe to administer it in every case, I cannot say, time alone must test that matter; and I think those cases, (if any,) where exceptions should be made, will at once suggest themselves to the mind of every skilful and observing practitioner.

4th. If all the cases of sickness, fainting, languor and debility, and subsequent confinement, which have occurred under the practice of every experienced dental surgeon, in connection with his operations, were published in detail, and hawked about in every newspaper, it would make a formidable list. And if those almost every-day occurrences were to happen in connection with the use of the Letheon, it would ring through the nation.

But I have already extended this paper to a greater length than I had intended. I think I have seen a disposition to forestall public sentiment in regard to this matter, and believing that one well authenticated fact is worth a thousand speculations, I have concluded to communicate the above experiments, and, in the language of one of olden time, "to show my opinion." And now I say, if this thing cannot stand the test of *just* and *faithful* experiment, let it go to the shades, with the strange fancies which its wonderful power is capable of conjuring up from the human brain. There is one thing more, however, in connection with this subject, which is worthy of attention, to wit: a spurious article, which is vended for the true "Letheon." Many are using such articles, to my knowledge, and, of course, any mischief which may arise from their use, will all be tacked on to the invention of Dr. Morton; and thus, like a genuine scape-goat, it must bear the sins of others.

NORWALK, CT., *Feb.* 12*th*, 1847.

